# Effectiveness and cost-effectiveness of an awareness campaign for colorectal cancer: a mathematical modeling study

**DOI:** 10.1007/s10552-014-0366-6

**Published:** 2014-03-29

**Authors:** Sophie Whyte, Susan Harnan

**Affiliations:** University of Sheffield, Sheffield, UK

**Keywords:** Colorectal cancer, Awareness campaign, Early diagnosis, Cost-effectiveness

## Abstract

**Background:**

A campaign to increase the awareness of the signs and symptoms of colorectal cancer (CRC) and encourage self-presentation to a GP was piloted in two regions of England in 2011. Short-term data from the pilot evaluation on campaign cost and changes in GP attendances/referrals, CRC incidence, and CRC screening uptake were available. The objective was to estimate the effectiveness and cost-effectiveness of a CRC awareness campaign by using a mathematical model which extrapolates short-term outcomes to predict long-term impacts on cancer mortality, quality-adjusted life-years (QALYs), and costs.

**Methods:**

A mathematical model representing England (aged 30+) for a lifetime horizon was developed. Long-term changes to cancer incidence, cancer stage distribution, cancer mortality, and QALYs were estimated. Costs were estimated incorporating costs associated with delivering the campaign, additional GP attendances, and changes in CRC treatment.

**Results:**

Data from the pilot campaign suggested that the awareness campaign caused a 1-month 10 % increase in presentation rates. Based on this, the model predicted the campaign to cost £5.5 million, prevent 66 CRC deaths and gain 404 QALYs. The incremental cost-effectiveness ratio compared to “no campaign” was £13,496 per QALY. Results were sensitive to the magnitude and duration of the increase in presentation rates and to disease stage.

**Conclusions:**

The effectiveness and cost-effectiveness of a cancer awareness campaign can be estimated based on short-term data. Such predictions will aid policy makers in prioritizing between cancer control strategies. Future cost-effectiveness studies would benefit from campaign evaluations reporting as follows: data completeness, duration of impact, impact on emergency presentations, and comparison with non-intervention regions.

## What is already known on this subject

Numerous primary studies have provided evidence about the impact of colorectal cancer awareness campaigns on public knowledge of campaign, knowledge of signs and symptoms, attitudes toward disease and treatment and behavior in terms of presentation to a GP, and uptake of screening. Until now, only one interim analysis of a primary study has provided evidence on effectiveness in terms of “change in cancer incidence” and no studies have reported data on mortality or cost-effectiveness.

## What this study adds

Our study demonstrates that it is possible to use a mathematical model in combination with short-term data from an awareness campaign to predict effectiveness (change in incidence and mortality) and cost-effectiveness (cost per quality-adjusted life-year).

Our study predicts that a national colorectal cancer awareness campaign in England would prevent 66 cancer deaths (based on data from the pilot awareness campaign).

Our study highlights key outcomes to report in the evaluation of future awareness campaigns, for example duration of campaign impact.

## Introduction

Cancer survival rates in England are poor compared to several other European countries, and there is increasing recognition that a considerable proportion of these avoidable deaths relate to late diagnosis [1, 2]. A National Awareness and Early Diagnosis Initiative (NAEDI) has been established in England as part of the Government’s strategy to improve cancer outcomes with one work stream, specifically focussing on raising public awareness and promoting earlier presentation by patients [3].

**Fig. 1:**
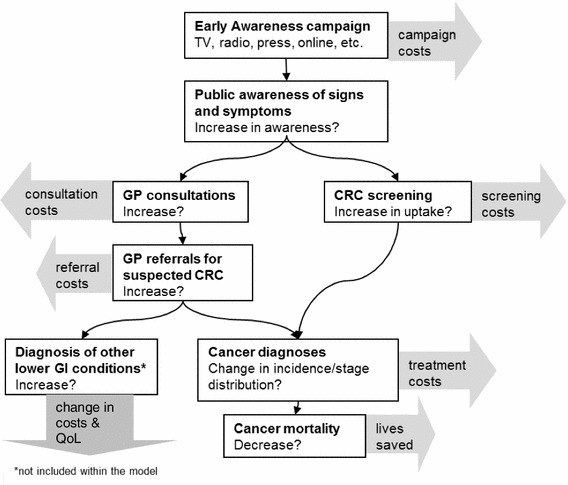
Potential effects of an early awareness campaign for colorectal cancer (CRC)

The National Cancer Action Team has been running a series of cancer awareness campaigns since 2009. The primary aim of cancer awareness campaigns is earlier presentation of symptomatic cancers through improved public knowledge of the symptoms [4]. Earlier presentation can result in cancers being diagnosed in earlier stages which may be associated with better survival and reduced treatment costs. However, a campaign may also lead to increased numbers of GP attendances by the “worried well”; indeed some critics assert that the campaigns will “undo years of work persuading patients with minor ailments to stay at home” [5]. Figure 1 summarizes the potential impacts of a colorectal cancer (CRC) awareness campaign.

A systematic review of available evidence on CRC awareness campaigns demonstrated that most studies focused on short-term outcomes such as “change in awareness” or “change in behavior” rather than longer-term outcomes such as “change in cancer incidence or mortality” [6]. Existing studies do not provide evidence of the mortality reduction associated with cancer awareness campaigns, few costs are reported, and no estimates of cost-effectiveness are available [6]. Collection of data on CRC mortality reduction following an awareness campaign would actually be unfeasible to collect as: (1) a long time-frame would be required; (2) a very large population would be required to demonstrate a statistically significant small change; and (3) it may be difficult to prove that any change can be attributed to the campaign rather than other factors.

In January 2011, a CRC “signs and symptoms” campaign was piloted in two regions of England and an evaluation was produced by the Department of Health [7]. We present estimates of the effectiveness and cost-effectiveness of a CRC awareness campaign which are generated using a mathematical model together with short-term data from this pilot campaign. Effectiveness is measured in terms of change in CRC incidence, CRC mortality, and quality-adjusted life-years (QALYs). Cost-effectiveness is measured as cost per QALY, incorporating the cost of the campaign, the cost of additional GP attendances, any change in CRC treatment costs, and QALYs accrued. The estimates provide an improved understanding of the benefits of such a campaign, which can be used to inform policy decisions around the selection of initiatives for the prevention of cancer morbidity and mortality.

## Methods

### Pilot colorectal cancer awareness campaign

**Fig. 2:**
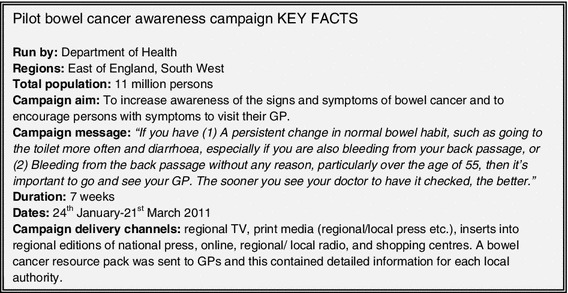
Pilot bowel cancer awareness campaign, key facts

Figure 2 summarizes the key facts relating to the pilot CRC awareness campaign. Data from the pilot campaign were available from the pilot evaluation report (March 2012) [7]. The pilot evaluation report included campaign running costs and data describing changes in GP attendances/referrals, CRC incidence, and CRC screening uptake. More recent, cancer incidence data were obtained from the South West Public Health Observatory and the Eastern Cancer Registration and Information Centre (October 2012) [8, 9]. Data on CRC detected at screening and screening uptake rates were also obtained from the NHS Bowel Cancer Screening Programme (BCSP) [10]. The data were analyzed to determine the magnitude and duration of the short-term impacts of the campaign, and this informed the mathematical model. The data illustrated an increase in the number of GP attendances, secondary care appointments, colonoscopy activity, and CRC incidence, which could be attributed to the campaign. A summary of the pilot campaign data used to inform the model is provided in Table 1. A detailed description of the data and modeling assumptions for the main pilot outcomes is provided below.

**Table 1:** Summary of data from the pilot campaign used in the modeling

	Data observed from pilot campaign	Base case assumption in model	Scenario analyses
GP attendances	700 increase over 3-month period (532 increase if diarrhea included as a symptom) Equivalent to 60,000–80,000 nationally.	70,000 more attendances nationally over 3-month period Assumed 50 % “additional” & 50 % “earlier”	Assumed 90 % “additional” & 10 % “earlier”
GP referrals	1956 increase in referrals over 5-month period (+28 %)	17,519 additional referrals nationally Assumed 50 % “additional”& 50 % “earlier”	Assumed 90 % “additional” & 10 % “earlier”
CRC incidence	7–11 % increase in incidence for 1 month	10 % increase in presentation rates for 1 month	5–20 % magnitude 1 to 6-month duration
CRC incidence stage distribution	Numbers too small to draw any conclusions	Campaign assumed to have the same proportional effect on presentation rates for each CRC stage.	Short-term increase in incidence only consists of Dukes’ stages C & D
CRC screening uptake	No significant change which could be attributed to the campaign	Assume screening uptake unaffected by campaign	Exploratory analysis undertaken
Cost of running campaign	£5 million	£5 million	–

### GP attendances

Data on the number of GP attendances with symptoms associated with CRC were available for a sample of practices for the period January 2010 to April 2011. During the 3-month period February to April 2011, GP attendances for the three symptoms rectal bleed, loose stools, and change in bowel habit increased by 700 (+62 %) and the increase was 532 (+20 %) if diarrhea was also included as a symptom. This would correspond to a further 60,000–80,000 GP attendance on a national scale. No change in the gender or age distribution of patients was evident. The increase in GP attendances was associated with considerable uncertainty due to large variations between practices and a possible change in symptom coding during the period of data collection. No data were collected for GP attendances following April 2011, so the duration of the effect of the campaign is uncertain. In the model, it was assumed that a national campaign would result in a further 70,000 GP attendance. There were no data to indicate what proportion of the increase in attendances were “additional” as opposed to “earlier”, so 50 % was assumed with 90 % considered in a sensitivity analysis.

### GP referrals

Data on the number of 2-week wait referrals from GP to secondary care with suspicion of lower gastrointestinal cancer was available for the months February to June for 2010 and 2011. The number of referrals was seen to increase by 1956 (+28 %) from 2010 to 2011 (corresponding to a further 17,519 on a national scale). As no data on the number of GP referrals for the period after June 2011 were available, the duration of the effect of the campaign is uncertain. There was evidence of an increase in colonoscopy demand and activity during the period February to June 2011 when compared to the previous year. The increase was estimated to be approximately 3,400 additional colonoscopies. There were no data to indicate what proportion of the increase in referrals were “additional” as opposed to “earlier”, so 50 % was assumed with 90 % considered in a sensitivity analysis.

### CRC incidence

**Fig. 3:**
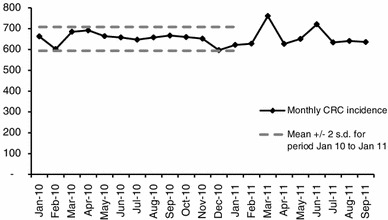
Colorectal cancer incidence in the East of England and southwest regions combined (data extract October 2012)

CRC has two possible routes of diagnosis: via the national screening program (10 % diagnosed via this route in 2010) or via symptomatic or chance presentation [10]. Data on monthly CRC incidence for the two pilot regions were available for the period January 2010 to September 2011. These data are presented in Fig. 3. As the campaign started at the end of January 2011, it was assumed that no change in incidence would be expected until March 2011 due to the likely time delay between making a GP appointment and receiving a diagnosis. A *t* test was undertaken to see if the cancer incidence observed in March 2011 was statistically significantly different compared to the proceeding period (January 2010 to February 2011). The pooled data set for the two regions had a *p*-value of less than 0.005, suggesting that an increase did occur in March 2011. The incidence for March 2011 was 11 % higher than that seen in March 2010 and 7 % higher than the mean +2sd for the period January 2011 to January 2012. No significant increase in incidence was observed for the period April 2011 onwards, using a threshold *p*-value of 0.01. Data on screen-detected CRC did not show any relationship with the awareness campaign [10]. The modeling assumes that the pilot campaign led to an increase in symptomatic detected incidence of 10 % for a period of 1 month only. Data on CRC incidence by Dukes’ stage at diagnosis involved very small numbers, so it was not possible to draw any significant conclusions regarding the impact of the campaign on the stage distribution.

There is considerable uncertainty surrounding the assumption that the pilot campaign caused an increase in incidence for the following three reasons. When the data from the individual regions were considered separately, there was more uncertainty: the *p*-values were 0.109 for the southwest data, 0.001 for the East of England data and 0.002 for the pooled data set. The analysis assumed that similar incidence would be expected in 2010 and 2011, but no data from a region not participating in the pilot were available to test this assumption. Monthly variations in incidence may occur as a result of factors such as different length months, different numbers of clinics, and different numbers of working days.

### CRC screening uptake

Data on uptake of screening during the period January 2010 to November 2011 were available from the BCSP for the two regions covered by the pilot [10]. An increase in overall uptake was observed during the campaign period; however, further data analysis suggests, this may not be due to the campaign as the increase occurred before the start of the campaign (from December 2010 to March 2011) [6]. Hence, no significant change in screening uptake which could be clearly attributed to the campaign was observed.

### Costs

The Department of Health provided the total cost of running the pilot campaign which was £1.6 million (£0.22 per person aged 30 or over) [7]. The budgeted cost for the national campaign (run in January 2012) was £4.5 million (£0.14 per person aged 30 or over). As this analysis will make predictions relating to a national campaign, a cost of £0.14 per person was used. The model also includes costs associated with additional GP attendances, CRC treatment costs, and CRC screening costs. Details of the costs used and their sources are provided in Tables 2 and 3.

**Table 2:** Model parameters associated with the awareness campaign

Awareness campaign parameters	Mean	Source
Increased presentation rates stage A	10 %	Pilot campaign data [7]
Increased presentation rates stage B	10 %	Pilot campaign data [7]
Increased presentation rates stage C	10 %	Pilot campaign data [7]
Increased presentation rates stage D	10 %	Pilot campaign data [7]
Duration of increase in presentation rates (months)	1	Pilot campaign data [7]
Increased screening uptake rate	0	NHS cancer screening 2012
Cost of campaign per person	£0.14	Department of Health 2012
Cost of GP visit (12 min consultation)	£36	Curtis 2010
Average cost of secondary care attendance for suspected lower GI cancer	£200	Costs from NHS reference costs 2011, probabilities from Tappenden [24]
Additional GP attendances per person	0.0014	Pilot campaign data [7]
Additional secondary care appointments per person	1.52606E−05	Pilot campaign data [7]
Proportion of additional visits which are extra	0.5	Assumption
Cost of additional GP and secondary care attendances	£0.026	Calculated from other parameters

**Table 3:** Parameters associated with CRC natural history and screening model

	Mean	Source
Screening participation and harm parameters		
FOBT participation for each screening round	0.54	NHS BCSP data [25]
Proportion completing at least one FOBT screening round	0.63	NHS BCSP data [25]
FOBT participation for a round for those who comply with at least one FOBT	0.85	Calculated from above parameters
COL follow-up compliance FOBT screening	0.79	NHS BCSP data [25]
COL surveillance compliance	0.83	NHS BCSP data [25]
COL (without polypectomy) perforation rate	0.0 %	FS UK screening trial data [26]
COL (with polypectomy) perforation rate	0.3 %	Bowel cancer screening pilot 2nd round evaluation [27]
COL Probability of death following perforation	5.2 %	Gatto et al. [28]
COL probability of hospitalization for bleeding	0.3 %	FS UK screening trial data [26]
Health-related quality of life parameters		
Utility value cancer free	0.80	Ara et al. [29]
Utility value CRC	0.70	Ara et al. [29]
Resource use parameters		
gFOBT mean number of tests completed	1.08	Assumption details in [14]
COL repeat test rate	0.07	NHS BCSP data [25]
Cost of gFOBT screen (non-compliers)	£2.03	Southern Hub screening costings model [14]
Cost of gFOBT screen (normal result)	£3.36	Southern Hub screening costings model [14]
Cost of gFOBT screen (positive result)	£11.94	Southern Hub screening costings model [14]
Cost of COL (without polypectomy)	£563	NHS ref costs, screening centre estimates [14]
Cost of COL (with polypectomy)	£563	NHS ref costs, screening centre estimates [14]
Cost of treating bowel perforation (major surgery)	£5,089	NHS reference costs [14]
Cost of admittance for bleeding (overnight stay on medical ward)	£278	NHS reference costs [14]
Pathology cost for adenoma/cancer	£26	NHS reference costs 08/09, histopathology [14]
Cost of treating colorectal cancer, Dukes’ stage A	£1,320–£8,375	Ranges presented reflect variation according to age at diagnosis. Generated using model from Tappenden [24]
Cost of treating colorectal cancer, Dukes’ stage B	£1,479–£8,362	
Cost of treating colorectal cancer, Dukes’ stage C	£1,493–£13,862	
Cost of treating colorectal cancer, Dukes’ stage D	£772–£11,198	
Test characteristics		
gFOBT sensitivity for LR adenomas	0.01	Model calibration [11]
gFOBT sensitivity for HR adenomas	0.12	Model calibration [11]
gFOBT sensitivity for CRC	0.24	Model calibration [11]
gFOBT specificity age 50	0.99	Model calibration [11]
gFOBT specificity age 70	0.97	Model calibration [11]
COL sensitivity for LR adenomas	0.77	Van Rijn et al. [30]
COL sensitivity for HR adenomas	0.98	Van Rijn et al. [30]
COL sensitivity for CRC	0.98	Bressler et al. [31]
COL specificity	1.00	Assumption due to nature of the test
Natural history parameters		
Normal epithelium to LR adenomas–age 30	0.021	Model calibration [11]
Normal epithelium to LR adenomas–age 50	0.020	Model calibration [11]
Normal epithelium to LR adenomas–age 70	0.045	Model calibration [11]
Normal epithelium to LR adenomas–age 100	0.011	Model calibration [11]
LR adenomas to high-risk adenomas–age 30	0.009	Model calibration [11]
LR adenomas to high-risk adenomas–age 50	0.008	Model calibration [11]
LR adenomas to high-risk adenomas–age 70	0.008	Model calibration [11]
LR adenomas to high-risk adenomas–age 100	0.004	Model calibration [11]
HR adenomas to Dukes’ A CRC–age 30	0.029	Model calibration [11]
HR adenomas to Dukes’ A CRC–age 50	0.025	Model calibration [11]
HR adenomas to Dukes’ A CRC–age 70	0.054	Model calibration [11]
HR adenomas to Dukes’ A CRC–age 100	0.115	Model calibration [11]
Normal epithelium to CRC Dukes’ A	0.00004	Model calibration [11]
Preclinical CRC: Dukes’ Stage A to B	0.51	Model calibration [11]
Preclinical CRC: Dukes’ Stage B to C	0.69	Model calibration [11]
Preclinical CRC: Dukes’ Stage C to D	0.71	Model calibration [11]
Symptomatic presentation with CRC Dukes’ A	0.04	Model calibration [11]
Symptomatic presentation with CRC Dukes’ B	0.18	Model calibration [11]
Symptomatic presentation with CRC Dukes’ C	0.37	Model calibration [11]
Symptomatic presentation with CRC Dukes’ D	0.74	Model calibration [11]
Proportion of cancer incidence classified as proximal	0.38	Cancer Registrations 2007, England [32] ]
Average number of adenomas present in patient with at least one adenoma	1.90	Winawer et al. [3, 33]
Proportion of advanced adenomas classified as HR adenomas	0.75	FS UK screening trial data [26]

### Mathematical model

**Fig. 4:**
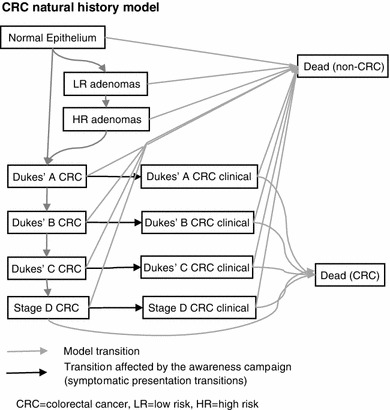
Natural History model diagram

An existing mathematical model for CRC screening was adapted for this project [11]. The model captures the natural history of CRC by representing the progression of pre-cancerous lesions (adenomas) to cancer and the progression of cancer through the Dukes’ stages. The model also represents the current CRC screening program of a biennial guaiac faecal occult blood test (gFOBT), follow-up of gFOBT positives with colonoscopy, and colonoscopic surveillance of high-risk individuals. The model has a state-transition structure and is built in Microsoft Excel with Visual Basic macros. Figure 4 illustrates the states and transitions included within the model. Uncertain model parameters are estimated using a process of model calibration, which is described in detail elsewhere [12]. Details of the natural history transition probabilities used in the model are provided in Table 3. The model takes the perspective of the NHS with a life-time horizon.

The modeling assumes that the awareness campaign results in a change in the probability of a person with undiagnosed CRC presenting symptomatically at their GP (as highlighted in Fig. 4). This change in the symptomatic presentation probabilities is determined so that model predictions of the change in CRC incidence reflect those seen following the pilot campaign. The diagnosis of cancer through symptomatic or chance presentation (non-screen-detected incidence) is represented in the model using a transition probability that is dependent on the Dukes’ stage of the cancer at presentation. As the data on CRC incidence by stage were inconclusive, the campaign was assumed to have the same proportional effect on non-screen-detected incidence for each CRC stage; a 10 % increase for a period of one month. This means that the stage distribution of the increase in incidence was the same as the stage distribution of incidence in the absence of the campaign (Dukes’ stages A, B, C and D: 11, 25, 36, and 29 %, respectively). The base case assumes that the campaign results in an increase in both symptomatic presentation and chance detection. However, a sensitivity analysis considered a situation where the increase in incidence was just made up of Dukes’ stages C and D as these stages are more likely to be associated with symptoms. This represents a scenario in which the campaign changes the rate of symptomatic presentation, but not chance detection.

Even though the campaign was assumed to have the same proportional effect on the presentation rates for CRC regardless of stage, the additional incidence due to the campaign corresponds to persons presenting earlier than they would have in the absence of the campaign and this earlier presentation results in a change in the stage distribution over the following years. The campaign was assumed to have the same effect on presentation rates for all age groups. The model responds to these adjusted symptomatic presentation probabilities by predicting associated changes in time to diagnosis, stage of diagnosis, and CRC mortality due to the campaign.

The additional colonoscopy activity caused by the campaign will increase adverse events associated with colonoscopy such as bowel perforations. The increase in polypectomies may also prevent some cases of CRC. The negative impact of additional colonoscopies (such as bleeding and bowel perforations) may be offset by the prevention of CRC through the removal of HR adenomas, so these were considered within a scenario analysis.

Model predictions were generated to reflect a national campaign as this was thought to be of most relevance for policy makers. Model predictions for no awareness campaign and an awareness campaign were produced. Predictions reflect changes in costs and QALYs for the lifetime of the entire current population of England aged over 30 (33 million persons). Predicted total costs were broken down to include the following: campaign costs, CRC treatment costs, and costs associated with additional GP attendances and referrals. Total QALYs, changes in cancer incidence, cancer stage distribution, and cancer mortality were also estimated. Future costs and QALYs were discounted at a rate of 3.5 % per annum in line with current NICE recommendations [13]. Net monetary benefit was calculated using a willingness-to-pay threshold of £20 K per QALY. Cost-effectiveness was reported in terms of cost per QALY saved.

Sensitivity analyses were undertaken for the following model parameters: the magnitude, duration and stage distribution of the short-term increase in incidence due to the campaign, and the proportion of the increase in GP attendances, which were additional. The model also allowed comparison between the potential benefits associated with an awareness campaign and an intervention designed to increase screening uptake. Probabilistic sensitivity analyses were undertaken, sampling 1,000 parameter sets to simultaneously explore the impact of uncertainty in disease natural history parameters, test characteristics, costs, and utility parameters. Distributions for parameters are described in the Whyte et al. 2011 report [14].

## Results

Model predictions were generated for an awareness campaign causing an increase in presentation rates of 10 % for 1 month. Table 4 shows model predictions broken down to include incidence by stage and diagnosis route (screen versus symptomatic detection) and the different components of associated costs. A total cost of £5.5 million is predicted, which comprises campaign costs (£4.5 million), additional GP consultation costs (£806 K), additional GP referrals (£50 K), and increased cancer treatment costs (£95 K).

**Table 4:** Model predictions for a CRC awareness campaign resulting in a 10 % increase in presentation rates for a period of 1 month

Model predictions for the current population of England evaluated over a lifetime: Change compared to “No awareness campaign”		
Outcome	Mean (from deterministic analysis)	95 percentiles from probabilistic sensitivity analysis*
CRC incidence–symptomatic presentation Dukes’ Stage A	26	(26, 28)
B	52	(49, 53)
C	33	(25, 38)
D	−92	(−96, −79)
CRC incidence–symptomatic presentation TOTAL	20	(19, 24)
CRC incidence screen/surveillance detected Dukes’ Stage A	−0	(0, 0)
B	−1	(−2, −1)
C	−2	(−3, −2)
D	−2	(−3, −1)
CRC incidence–screening/surveillance detected TOTAL	−5	(−7, −5)
CRC-specific deaths	−66	(−69, −56)
Deaths with undiagnosed CRC	−14	(−17, −14)
Total costs related to screening (discounted)	−£3,407	(−4,498, −2,855)
Cancer management (inc. pathology) costs (discounted)	£94,443	(88,853, 116,287)
Cost of additional GP consultations/referrals (discounted)	£855,716	(855,716, 855,716)
Cost of awareness campaign (discounted)	£4,499,995	(4,499,995, 4,499,995)
Total cost (discounted)	£5,446,745	(5,441,070, 5,468,342)
Total life-years gained (undiscounted)	991	(833, 1,041)
Total life-years gained (discounted)	622	(516, 657)
Total QALYs gained (discounted)	404	(322, 439)
ICER	£13,496	(12,407, 16,893)
NMB	£2,624,770	(1,001,887, 3,330,998

A campaign is predicted to prevent 66 deaths from CRC and generate an additional 404 QALYs. It is estimated to cause an increase in the number of cases of Dukes’ stages A–C presenting symptomatically and a decrease in the number of cases of stage D. Overall, an increase in symptomatic presentation and a small decrease in screen/surveillance detected cases is predicted. A significant reduction in CRC-specific deaths was seen, which was due to the reduction in the number of cases of CRC presenting in stage D. This reduction in deaths corresponds to an increase in QALYs gained. There is also a small decrease in the number of persons dying with undiagnosed CRC. The incremental cost-effectiveness ratio (ICER) for the awareness campaign was £13,496 per QALY gained compared to “no campaign” giving a net monetary benefit (NMB) of £2.6 million (with a willingness-to-pay threshold of £20 K per QALY).

**Fig. 5:**
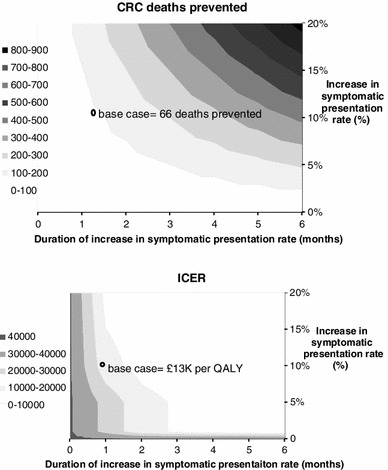
Two-way sensitivity analyses to explore uncertainty in the increase in presentation rates caused by the campaign: varying the duration and magnitude

**Fig. 6:**
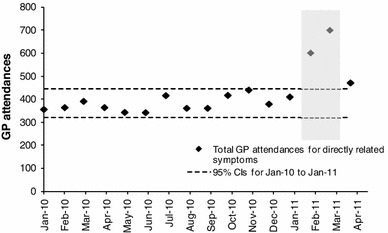
GP attendances associated with CRC symptoms

**Fig. 7:**
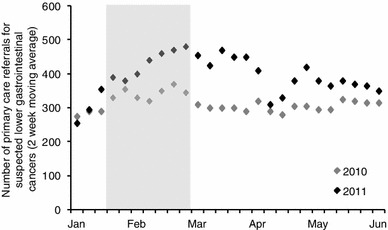
Referrals from primary care for suspected lower GI cancer

Table 5 and Fig. 5 show the results of sensitivity analyses on the increase in presentation rates parameters. Figure 5 shows the predicted QALY gain for a two-way sensitivity analyses which varied the duration and magnitude of the increase in presentation rates caused by the campaign. The analysis demonstrates that the results are highly sensitive to these two parameters with predicted mortality reductions ranging from 66 (1 month increase in 10 %) to over 800 (6 month increase in 20 %). Similarly, QALY gain ranged from 202 to 5283 and the ICER ranged from £1 K to £27 K per QALY. An analysis in which the increase in presentation rates was restricted to Dukes’ stages C and D was undertaken. In this analysis, an increase in Dukes’ stages C and D presentation rates of 15 % was applied as this corresponds to an increase in all stage incidence of 10 %. This analysis showed lower cost-effectiveness resulting in a gain of 293 QALYs and an ICER of £21 K. The one-way sensitivity analyses demonstrated that the uncertainty surrounding the increase in presentation rate parameters had a big impact on the effectiveness of the campaign. A scenario analysis in which 90 % of the increase in GP attendances/referrals was assumed to be additional as opposed to earlier resulted in only a small increase in the ICER associated with the campaign. Probabilistic sensitivity analyses demonstrated that the uncertainty in natural history parameters, test characteristics, cost, and utility parameters rates had less influence on model predictions than variations in the campaign effect on presentation rates (Figs. 6, 7).

**Table 5:** Sensitivity analyses to explore uncertainty in the increase in presentation rates caused by the campaign: varying the duration, magnitude, and stage distribution

	CRC deaths prevented				QALY gain			ICER		
	Magntiude of change in symptomatic presentation rate (% increase)									
		5 %	10 %	20 %	5 %	10 %	20 %	5 %	10 %	20 %
Campaign causes increase in symptomatic presentation rate for all stages of colorectal cancer										
Duration of change in symptomatic presentation rate (months)	1	33	66	131	202	404	807	£26,767	£13,496	£6,861
	3	101	202	403	622	1,243	2,487	£8,843	£4,536	£2,383
	6	210	419	838	1,296	2,592	5,183	£4,368	£2,301	£1,268
Campaign causes increase in symptomatic presentation rate for Dukes’ stages C and D colorectal cancer										
	1	14	28	57	98	196	391	£55,210	£27,826	£14,135
	3	44	89	177	306	611	1,223	£17,965	£9,205	£4,825
	6	94	189	378	651	1,302	2,605	£8,672	£4,560	£2,504

Colonoscopy is associated with a risk of hospitalization for bleeding of 0.03 % and a negligible risk of perforation (unless polypectomy is performed). Data on the number of GP referrals which lead to colonoscopy were not available, but in an extreme scenario where 100 % receive colonoscopy, the campaign would be predicted to result in 50 cases of hospitalization due to bleeding at a cost of £14 K.

There is a considerable body of evidence demonstrating the efficacy of campaigns designed to increase screening uptake [15]. Investment in such campaigns is another route toward reducing CRC mortality. We considered the two groups: “screening never attenders” and “screening sometimes attenders” as defined in the screening reappraisal paper [16]. An exploratory analysis estimated that a reduction in the number of persons in the group “screening never attenders” by 0.09 % (from 36.55 to 36.52 %) would result in the same gain in QALYs as was predicted by the awareness campaign.

## Discussion

This study demonstrates that it is possible to use a mathematical model together with short-term data from a pilot CRC awareness campaign to make predictions of both effectiveness and cost-effectiveness. The campaign was predicted to reduce CRC mortality and have an ICER of less than £20,000. However, scenario analyses indicated that results were highly sensitive to the duration, magnitude, and stage distribution of the increase in presentation rates due to the campaign. The model structure also allows a comparison of an early awareness campaign and a campaign designed to increase screening uptake. The study also identified priorities for future awareness campaign evaluations, which are described below.

The data obtained from the pilot campaign should accurately reflect the use of such a campaign in a UK NHS setting. Hence, this study is of direct relevance for policy making within the UK. The main weakness of this study is the limited evidence available on duration of the impact of the awareness campaign. However, the impact of the assumption on duration was explored within a sensitivity analysis. A weakness of this analysis is that it was not possible to represent all possible impacts of an awareness campaign within the modeling. In 2007, approximately a quarter of cancer cases in the UK were diagnosed through emergency admission to hospital [17]. No data were available on the change in presentation mode (GP versus emergency presentation) as a result of the campaign. Hence, any potential cost savings as a result of preventing emergency presentations of CRC was not incorporated within the modeling. The awareness campaign is designed to increase presentation rates for symptoms associated with CRC, which may result in the earlier diagnosis of other lower GI conditions such as Crohns disease, ulcerative colitis, inflammatory bowel disease, and piles. No data were available on changes in diagnosis rates for other conditions, so any associated costs or QALY differences are not represented by the model.

This study predicts the effectiveness and cost-effectiveness based on data from an awareness campaign run in 2011. The benefits of such a campaign may well be subject to change over time as general public awareness changes. For example, a US study examined the number of diagnoses made in the month after National Breast Cancer Awareness Month (NBCAM) saw an increase in diagnoses during the period when breast cancer advocacy was expanding rapidly into a nationwide movement, but no significant change during earlier periods when breast cancer advocacy was still a grassroots movement, and in later periods, when breast cancer advocacy had become a well-established nationwide cause [18].

Comparison with results from other studies was not possible as no similar studies which predict the cost-effectiveness of a CRC awareness campaign were identified by the literature review. Prior studies have estimated the cost-effectiveness of patient-directed interventions for CRC screening (such as a mailed educational reminder for FOBT screening) are associated with costs ranging from $15 to $5842 [19]. However, these cannot be compared as they do not report CRC deaths avoided or cost per QALY.

### Implications for policy

Prioritizing between different cancer mortality reduction strategies is a great challenge for policy makers. Interventions with the potential to reduce CRC mortality include the following: awareness campaigns, improvements to the screening program (e.g., different diagnostic tools [16, 20]), measures to improve uptake [15], interventions to change lifestyle and risk factors [21], interventions to improve diagnosis (e.g., cancer prediction models [22]), and new cancer treatments. This study provides new evidence on the effectiveness and cost-effectiveness of a CRC awareness campaign to help inform such decisions. The evaluation of cost-effectiveness as cost per QALY allows comparison with other interventions designed to reduced cancer mortality. For example, a cost-effectiveness analysis of chemoprevention report an ICER of £23 K for aspirin chemoprevention in the general population compared to screening alone [23]. This study highlights the potential to compare the cost-effectiveness of a CRC awareness campaign and a campaign designed to improve screening uptake. The availability of data on the cost and effectiveness of a campaign to improve screening uptake in England would allow such a comparison.

### Future research

The data available from the pilot campaign which was used to generate predictions of efficacy and cost-effectiveness was associated with limitations and considerable uncertainty. A priority for future research is to co-ordinate and maximize the evaluation and dissemination of efforts that have already been made to increase cancer awareness. In particular, comparison with non-intervention regions and clear reporting of completeness of data and potential data limitations are essential. To establish the potential effectiveness and cost-effectiveness of such a campaign information on “duration of effect of campaign,” “effect of campaign on CRC incidence,” “effect of campaign on emergency presentation rates,” and “effect of campaign by age” are of importance. In addition, data on differential diagnoses costs associated with emergency presentation versus two-week wait referrals would be of use for future modeling exercises. The exploratory analysis into the cost-effectiveness of a campaign to increase screening uptake could be developed further if data on the cost and effects associated with an actual campaign from the UK were available.
